# Effect of adding soluble viscous fibers to diets containing coarse and finely ground insoluble fibers on digesta transit behavior and nutrient digestibility in broiler chickens

**DOI:** 10.1016/j.psj.2024.103487

**Published:** 2024-01-30

**Authors:** Sebastián Dorado-Montenegro, Mochammad F. Habibi, Walter J.J. Gerrits, Sonja de Vries

**Affiliations:** ⁎ Animal Nutrition Group, Wageningen University & Research, Wageningen, Gelderland, 6700 The Netherlands; † Escuela de Zootecnia, Universidad de Costa Rica, San José, Costa Rica, 2060 San José

**Keywords:** arabinoxylans, markers, mean retention time, particle size, soybean hulls

## Abstract

This paper aimed to study the interactive effects of the addition of soluble arabinoxylans (**AX**) and the particle size (**PS**) of soybean hulls (**SBH**) on digesta mean retention time (**MRT**) and nutrient digestibility in broiler chickens. A total of 288 one-day old Ross 308 female chicks were assigned to 32 pens (9 birds/pen) and fed a commercial starter diet for 10 d. At 10 d of age, pens were assigned to 1 of 4 dietary treatments (8 pens/diet) containing 120 g/Kg coarse or fine SBH, with or without addition of 50 g/Kg of soluble wheat AX, substituting maize starch. Titanium dioxide (4 g/Kg) and cobalt-EDTA (1 g/Kg) were added as inert markers. Excreta were quantitatively collected from d 22 to 25. Gastrointestinal tract and digesta were collected on d 28, 29, or 30. Arabinoxylans reduced the weight of the gizzard relative to body weight (**RW**) by 0.07% units (*P* = 0.005), and increased ceca RW (0.28 vs. 0.34%, *P* < 0.001) and length (10.45 vs. 11.21 cm/Kg BW, *P* < 0.001). Arabinoxylans increased digesta MRT in the crop (solids/liquids: +12 min, *P* < 0.05), small intestine (solids/liquids: +17 min, *P* < 0.01), and hindgut (liquids: +77.5 min, *P* < 0.05); and reduced apparent ileal digestibility (**AID**) and apparent total tract retention (**ATTR**) of DM (−5.4 and −3.9%, *P* < 0.001, respectively) and starch (−1.35 and −0.7%, *P* < 0.001, respectively). Particle size of SBH only affected the ATTR of non-starch polysaccharides, presenting higher retention values with fine SBH (−4.3%-units, *P* = 0.034). The addition of AX reduced AID of N by 4.3%-units, only in presence of fine SBH (interaction, *P* < 0.05). In conclusion, arabinoxylans greatly influenced digestion in the chicken GIT, while PS of SBH had marginal effects. Arabinoxylans reduced AID of N only with fine SBH, suggesting coarse SBH counteracted AX effects on N digestion, speculatively by modifying digesta viscosity.

## INTRODUCTION

The increased use of by-products from agricultural and food industries in animal diets has stimulated extensive research to comprehend the impact of dietary fibers (**DF**) on digestive processes. Dietary fiber has the potential to influence the digestive physiology of monogastric animals by modulating the physicochemical properties of the digesta (Smits et al., 2000), digesta transit behavior along the gastrointestinal tract (**GIT**) (Svihus et al., 2002), and by serving as a substrate for microbial fermentation (reviewed by Williams et al., 2019). However, due to significant peculiarities in the chemical and structural characteristics of DF (Bach Knudsen, 1997), their effects on digestive processes and, ultimately, nutrient digestibility, may vary among sources, leading to either beneficial or detrimental outcomes for the animals.

In chickens, the addition of insoluble DF (**iDF**) has been shown to have positive effects on the development of the gizzard (Hetland et al., 2003; Sadeghi et al., 2020), consequently allowing a better grinding and mixing of digesta with digestive enzymes (reviewed by Svihus, 2011). Several studies have reported no effect or even improvements in nutrient digestibility and productive performance when iDF were added to the diets of broilers and laying hens, even though the degradation of iDF in poultry is negligible (Jørgensen et al., 1996; Hetland et al., 2003; González-Alvarado et al., 2007; Jiménez-Moreno et al., 2011; Kalmendal et al., 2011; Yokhana et al., 2016; Tejeda and Kim, 2020; Zhang et al., 2023). Furthermore, there is evidence that particle size (**PS**) of iDF is partially responsible for the iDF effects on gastrointestinal tract development (Jiménez-Moreno et al., 2010), digesta retention time (Moore, 1999), and nutrient digestibility (Donadelli et al., 2019; Pourazadi et al., 2020) in poultry. For instance, Sacranie et al. (2012) reported that adding 150 g/Kg of a 50:50 mixture of oat and barley hulls to broiler diets resulted in a fuller and larger gizzard, with birds fed the coarse hulls presenting the largest gizzards. Moreover, Hetland et al. (2005) observed that about 30% of coarse (unground) oat hulls were still found in the gizzard 48 h postfeeding, while 90% of fine (ground using a 0.5 mm sieve) oat hulls had passed the gizzard after 2 h.

Although iDF may be partly beneficial for broilers, the addition of soluble DF (**sDF**) has been mainly associated with negative effects. Several studies have reported that the addition of sDF increase digesta viscosity, modulates digesta retention time, and has negative effects on nutrient digestibility and productive performance (van der Klis and van Voorst, 1993; Van Der Klis et al., 1993; Saki et al., 2011).

However, there is limited understanding regarding how the addition of sDF may impact the digestion process in the presence of various PS of iDF, as well as the potential interactions between these factors. Since digesta consists of a combination of solid particles suspended in a liquid, alterations in viscosity, which is a natural property of liquids, may impact the digestive processes differently when particle size is modified. Therefore, the objective of this paper was to study the effects of adding soluble viscous fibers, in form of arabinoxylans (**AX**), to diets containing coarse versus finely ground insoluble fibers, in the form of soybean hulls (**SBH**), on digesta mean retention time (**MRT**), and nutrient digestibility in broiler chickens. We hypothesized that coarse iDF will prolong solid digesta MRT in the proximal GIT, and improve nutrient digestibility, while AX addition will prolong liquid digesta MRT and reduce nutrient digestibility. Moreover, the negative effect of viscous fibers over nutrient digestibility is expected to be reduced in the presence of fine PS of the iDF.

## MATERIAL AND METHODS

### Ethics Declaration

The experiment was approved by the National Central Committee of Animal Experiments (**CCD**) under the permit number AVD1040020197324, in accordance with the Dutch Act on Animal Experimentation and EU Directive 2010/63/EU; and performed at the research facility Carus, Wageningen University & Research, (Wageningen, The Netherlands).

### Animals and Housing

A total of 288 female one-day-old Ross 308 broiler chicks (initial body weight: 40.7 ± 3.22 g) were randomly distributed over 48 pens, and pens were assigned to 1 of 4 treatments following a randomized block design with a 2×2 factorial arrangement of treatments. Each treatment had 8 replicate pens of 9 birds each. Under controlled environmental conditions, each pen was equipped with plastic slatted floor covered with cardboard matting and wood shavings, 6 nipple drinkers, 1 hopper feeder, and 1 perch. Birds were vaccinated at 14 d of age against Newcastle disease.

### Dietary Treatments and Diets

During the first 10 d, all birds were fed a commercial pelleted maize-soybean based starter diet. From day 10 and until the end of the experiment (d 30), the pelleted experimental growing diets were offered (Table 1). A maize-based diet with soy protein concentrate, fish meal, potato protein, and 120 g/Kg of SBH was formulated to meet or exceed the requirements for growing broiler chickens (CVB, 2018; Aviagen, 2019). We formulated 4 dietary treatments, where SBH were included in coarse (**SBH-C**: unground; geometric mean diameter (**GMD**) 1134 μm ± 144 μm) or fine (**SBH-F**; ground with a 1 mm screen, GMD 459 μm ± 85 μm) form; and 50 g/Kg soluble wheat arabinoxylans (Naxus, BioActor BV, Maastricht, The Netherlands) were substituted for maize starch (**SBH-C-AX** and **SBH-F-AX**) or not (SBH-C and SBH-F). All the experimental diets contained 4 g/Kg of titanium dioxide (**TiO_2_**) as inert markers to represent the solid digesta phase and 1 g/Kg of cobalt-EDTA (**Co-EDTA**) to represent the liquid phase. Feed and water were available ad libitum during the whole experiment.

**Table 1:** Ingredient and nutritional composition of experimental diets fed to female growing broiler chickens from 10 to 30 d of age.^1^^,^^2^^,^^3^^,^^4^

Item	SBH-C / SBH-F	SBH-C-AX / SBH-F-AX
Ingredient composition (g/Kg)		
Maize	450.0	450.0
Maize Starch	175.1	126.1
Soy protein concentrate	126.0	126.0
Soybean hulls	120.1	120.1
Arabinoxylans	-	50.0
Sugar	28.4	28.4
Potato protein	1.5	1.5
Fish meal	24.8	24.8
Soybean oil	15.0	15.0
Potassium carbonate	3.9	3.9
Sodium bicarbonate	2.0	2.0
L-Lysine	5.0	5.0
D-Methionine	4.1	4.1
L-Threonine	2.7	2.7
L-Tryptophane	0.2	0.2
L-Isoleucine	1.2	1.2
L-Arginine	3.2	3.2
L-Leucine	0.5	0.5
L-Valine	2.4	2.4
Mineral and vitamin Premix	5.0	5.0
Monocalcium phosphate	11.7	11.7
Salt	1.2	1.2
Calcium carbonate	9.0	8.0
Cobalt-EDTA	1.0	1.0
Titanium dioxide	4.0	4.0
Polyethylene glycol	2.0	2.0
Total composition (g/Kg)	1000.0	1000.0
Calculated nutritional composition (g/Kg)		
Crude Protein	172.0	-
Dig Lys	11.1	-
Dig Thr	7.6	-
Dig Met + Cys	7.2	-
Ca	7.2	-
Available P	2.9	-
AME (kcal/Kg)	3033.0	-
Analysed nutritional composition (g/Kg)		
Dry matter	902.1	900.7
Crude Protein	171.8	180.7
Crude Fat	35.5	37.7
Crude ash	50.6	51.4
Total non-starch polysaccharides	116.5	139.6

1 Arabinoxylans (AX) (Naxus, BioActor BV, Maastricht, The Netherlands).

2 SBH-C: soybean hulls coarse; SBH-F: soybean hulls fine.

3 Premix provided per kilogram of diet: Vitamin A (retinyl acetate), 10.000 IU; Vitamin D_3_ (cholecalciferol), 2.500 IU; Vitamin E (dl-a-tocopherol), 50 mg; Vitamin K_3_ (menadione), 1.5 mg; Vitamin B_1_ (thiamin), 2.0 mg; Vitamin B_2_ (riboflavin), 7.5 mg; Vitamin B_6_ (pyridoxin-HCl), 3.5 mg; Vitamin B_12_ (cyanocobalamin), 20 µg; Niacin, 35 mg; D-pantothenic acid, 12 mg; Choline chloride, 460 mg; Folic acid, 1.0 mg; Biotin, 0.2 mg; Iron, 80 mg, as FeSo_4_; Copper, 12 mg, as CuSO_4_; Manganese, 85 mg, as MnO; Zinc, 60 mg, as ZnSO_4_; Iodate, 0.8 mg, as KJ; Selenium, 0.15 mg, as Na_2_SeO_3_.

4 Calculated nutritional composition based on the composition and nutritional value of feed materials (CVB, 2018). Digestible amino acids and metabolizable energy values were calculated based on values of standardized ileal digestibility and apparent metabolizable energy for broilers.

### Sampling and Data Collection

Birds were individually weighed on d 1, 10, 21, and immediately prior to being euthanized. Feed consumption per pen was measured at d 10, 21, and 25. On d 18, the cardboard matting and wood shavings were removed to prevent ingestion of bedding. From d 22 to 25, excreta were quantitatively collected and weighed per pen twice a day (at 8 am and 2 pm). A subsample of clean excreta was collected and frozen immediately in the freezer at –20°C. All the daily collected excreta subsamples from each pen during these 4 d were then homogenously mixed in a bucket and a portion was taken for the analysis of apparent total tract retention.

At d 28, 29, or 30, we euthanized 6 birds/pen (192 in total), according to a dissection schedule designed to obtain digesta flowing curves for the liquid and solid phase, after a pulse dose of markers (data not shown). An injection of 0.5 mL sodium pentobarbital (20% or 500 mg/mL) was applied to each bird at the base of the back edge of the skull. The whole gastrointestinal tract was removed, and divided into 7 segments (crop, proventriculus, gizzard, first half of small intestine (**SI**) until Meckel's diverticulum, second half of SI until ileo-ceca junction, ceca, and colon) using tie wraps. Each segment was weighed full and empty, and digestive contents were quantitatively collected and frozen at –20°C per bird. Moreover, the thickest part of the gizzard wall was measured with a caliper (mm), and the length of each cecum per bird was measured using a measuring tape. The measurement of GIT traits was conducted as supplementary data to provide further understanding of the potential effects of fibers on the digestion process.

The contents from the gizzard and proventriculus were combined and treated as a unified compartment (hereafter referred to as Stomach) for analysis of digesta MRT. Also, the MRT of ceca and colon was analyzed as one compartment (hereafter referred to as hindgut). The reasoning for this is the occurrence of antiperistatical movements in the passage of digesta between these organs (Duke, 1982), which invalidates the assumptions made for measuring digesta retention time using markers (de Vries and Gerrits, 2018). Crop and stomach samples were pooled and analyzed per pen, while SI, ceca, and colon samples were analyzed per bird (for another experiment). Subsamples were obtained from the distal SI and pooled per pen to analyze apparent ileal digestibility of nutrients. The rest of digesta content from the distal SI segment was then mixed with a subsample of digesta from the proximal SI, both with the same weight proportion, to create a representative sample of all the small intestine per bird.

During the experimental period, approximately 500 g of each experimental diet was randomly collected from different feeders at a single time point, and the samples were stored in sealed and labeled plastic bags at 4°C until their chemical composition was analyzed.

### Analyses and Calculations

*Particle Size Analysis.* Particle size distribution, geometric mean diameter **(GMD**), and geometric standard deviation (**GSD**) of SBH-C and SBH-F were analyzed in duplicate using dry sieving analysis (ASABE, 2008). Geometric mean diameter and GSD of the fibrous ingredients were calculated as described by Lyu et al. (2020).

*Water Binding Capacity***(WBC)*.*** Samples were mixed with deionized water inside a conical tube (50 ml) using a vortex (2500 rpm) for 5 s to ensure thorough mixing of the components. The WBC of fibers and diets was analyzed in duplicate based on Jacobs et al. (2015).

*Digesta Mean Retention Time***(MRT)*.*** Mean retention time of solid and liquid digesta was calculated using Ti and Co as inert markers. Under continuous dosing (steady state) condition, the marker input equals the output in each segment of the GIT. Thus, MRT was calculated using the following equation (de Vries and Gerrits, 2018):

$$\text{MRT}\;\left(\text{min}\right)\;=\;1440\times \frac{T_{sample}\times Q_{sample}}{T_{diet}\;\left(intake/day\right)}$$

where Tsample is the concentration of the marker Ti (g/Kg) or Co (mg/Kg) in the sample (digesta of each segment), Tdiet is the concentration of the marker in the feed, and Qsample is the quantity of the sample (Kg). To calculate the marker consumption, an average feed intake was calculated from the data collected from d 21 to 25 and extrapolated to the day of euthanasia for each bird. Digesta phase segregation (min) was calculated as the difference between the MRT of the digesta phases and presented as absolute values.

*Nutrient Digestibility and Retention.* Samples of ileal digesta and excreta were freeze-dried prior to analyses, diet samples were analyzed on as-fed basis. All samples were ground using a centrifugal mill with a 1 mm sieve (Retsch ZM 200). Feed, ileal digesta, and excreta were analyzed for dry matter (DM; ISO 6496:1999), starch (ISO 15914:^2004^), nitrogen (N; ISO 5983:^2005^), nonstarch polysaccharides (NSP; Englyst et al., 1994), Ti and Co (van Bussel et al., 2010). Feed were additionally analyzed for fat after acid hydrolysis (ISO 6492:^1999^) and ash (ISO 5984:^2002^). The apparent ileal digestibility (**AID**) and apparent total tract retention (**ATTR**) of DM, starch, nitrogen (**N**), and non-starch polysaccharides (**NSP**) were calculated using the following equation:

$$\text{AID}\;\text{or}\;\text{ATTR}\;\left(\%\right)\;=\;100\left[1-\left(\frac{T_{diet}}{T_{sample}}\right)\times \left(\frac{Nutrient_{sample}}{Nutrient_{diet}}\right)\right]$$

where T_diet_ is the concentration of Ti in the feed, and T_sample_ in the ileal digesta or in the excreta. Moreover, Nutrient_diet_ is the concentration of nutrient (DM, N, starch, NSP) in the feed and Nutrient_sample_ in the ileal digesta or in the excreta. All the units are in g/Kg.

### Statistical Analyses

Pen (6 birds/pen for all variables, except for ATTR, 9 birds/pen) was considered the experimental unit for all the analysis. Data were analyzed using a general linear model (PROC GLM, SAS 9.4) with GIT traits, digesta MRT, digesta phase segregation, and digestibility or retention values as dependent variables, and AX, PS, and their interaction as fixed effects. Model residuals were visually assessed to verify model assumptions. Crop relative weight (**RW**), proventriculus RW, and the MRT for both solid and liquid digesta in the crop and stomach did not meet the assumption of normal distribution of the model residuals. Hence, these parameters were logarithmic transformed, prior to statistical analyses. Differences among means were tested using type III least squares statistics, using Tukey adjustments for multiple comparisons. Data are presented as (back transformed) estimated means and pooled SEM. Differences among means were considered to be statistically significant when *P* < 0.05.

A Pearson's correlation analysis (PROC CORR, SAS 9.4) was conducted to study the correlations among DM and starch disappearance in the hindgut (ATTR-AID), NSP ATTR, ceca length, and ceca RW.

## RESULTS

The grinding procedure decreased the GMD of SBH by almost 2.5 times, reaching a contrast in particle size of 675 μm (1134 ± 144 μm vs. 459 ± 85 μm) (Table 2). Particles larger than 630 mm, accounting for approximately 95% of the total SBH-C, were reduced to only 20% of the total SBH-F.

**Table 2:** Particle size distribution (%), geometric mean diameter (GMD, μm), and geometric standard deviation (GSD, μm) of coarse and fine soybean hulls (SBH), measured using dry sieving method.^1^^,^^2^^,^^3^

Feed ingredient	Nominal sieve aperture, μm							GMD, μm	GSD, μm
	2500	1250	630	315	160	71	<71		
Soybean hulls coarse	0.75	39.15	55.59	2.79	0.46	0.37	0.99	1134	144
Soybean hulls fine	0.00	0.02	20.27	55.78	12.21	5.52	6.44	459	85

1 Coarse hulls offered unground.

2 Fine hulls ground in a hammermill with a screen size of 1.0 mm.

3 GMD (n) = 2 replicates.

### Gastrointestinal Tract Traits

The addition of AX reduced gizzard (1.42 vs. 1.36%, *P* = 0.005) and stomach (2.01 vs. 1.90%, *P* = 0.017) weight relative to body weight (Table 3). However, ceca weight (0.34 vs. 0.28%, *P* < 0.001), and ceca length relative to body weight (11.21 vs. 10.45%, *P* = 0.001) increased with the addition of AX. Particle size of the SBH did not have any impact on the GIT traits (*P* > 0.05) and no interactions between particle size and AX were observed.

**Table 3:** Effect of arabinoxylan (AX) addition (50 g/Kg) to diets with different particle size of soybean hulls (SBH) on body weight, feed intake, and gastrointestinal traits of broiler chickens measured at 28, 29, or 30 d of age.^1^^,^^2^^,^^3^

Particle size	Coarse		Fine		Pooled SEM	Particle size	AX	Particle size x AX
AX (g/Kg)	0	50	0	50				
Daily feed intake (g/d)	96 ± 4.2	96 ± 6.9	97 ± 3.6	96 ± 5.6	-	-	-	-
Body weight (g)	1360 ± 71.7	1404 ± 50.8	1323 ± 46.3	1347 ± 63.6	-	-	-	-
Relative weight (RW, %)								
Crop	0.27	0.29	0.29	0.29	0.011	0.263	0.706	0.554
Proventriculus	0.57	0.60	0.55	0.55	0.030	0.739	0.218	0.562
Gizzard	1.43	1.36	1.41	1.35	0.026	0.981	0.005	0.517
Stomach	2.01	1.89	2.01	1.90	0.046	0.862	0.017	0.989
Ceca	0.27	0.34	0.29	0.33	0.010	0.757	<0.001	0.357
Gizzard thickness (mm)	9.0	9.0	8.9	9.0	0.26	0.811	0.837	0.753
Ceca length (cm/Kg BW)	10.3	11.1	10.6	11.3	0.20	0.165	0.001	0.635

1 n = 8 replicate pens (6 birds/pen) for all treatments.

2 Stomach: Proventriculus + Gizzard.

3 Coarse: hulls offered unground; Fine: hulls ground in a hammermill with a screen size of 1.0 mm.

### Mean Retention Time of Digesta Phases along the Gastrointestinal Tract

The addition of AX in the diets resulted in an increase in MRT for both digesta solids and liquids (+12 min for both) in the crop (*P* < 0.05) (Table 4). Neither the particle size of SBH nor the addition of AX affected the MRT of solid or liquid digesta in the stomach (*P* > 0.05). Segregation of digesta phases in the stomach, however, tended to be reduced by SBH-F by 6 min (*P* = 0.061) compared with SBH-C.

**Table 4:** Effect of arabinoxylan (AX) addition (50 g/Kg) to diets with different particle size of soybean hulls (SBH) on the mean retention time (MRT, min) of the solid and liquid digesta phase in the crop, proventriculus and gizzard (stomach), small intestine, caeca and colon (hindgut) in broiler chickens, measured at 28, 29, or 30 d of age.^1^^,^^2^^,^^3^^,^^4^

Segment	Digesta fraction	Particle size	Coarse		Fine		Pooled SEM	Particle size	AX	Particle size x AX
		AX (g/Kg)	0	50	0	50				
Crop	Solids		16	30	18	29	4.8	0.819	0.014	0.691
	Liquids		15	29	17	27	4.5	0.899	0.010	0.745
	Phase segregation (min)		1	1	2	1	0.5	0.533	0.921	0.332
Stomach	Solids		77	75	72	59	8.6	0.218	0.375	0.504
	Liquids		45	45	44	40	4.9	0.509	0.763	0.640
	Phase segregation (min)		32	29	28	19	4.1	0.061	0.107	0.371
Small Intestine	Solids		235	247	221	243	6.0	0.142	0.007	0.422
	Liquids		200	218	190	216	5.4	0.264	<0.001	0.429
	Phase segregation (min)		34	29	31	27	3.0	0.340	0. 169	0.856
Hindgut	Solids		49	65	57	61	5.2	0.749	0.080	0.238
	Liquids		189	297	250	297	31.3	0.331	0.020	0.337
	Phase segregation (min)		140	232	193	237	30.0	0.338	0.032	0.425
Total GIT	Solids		380	428	380	399	18.1	0.429	0.077	0.436
	Liquids		451	594	509	587	35.2	0.478	0.004	0.357
	Phase segregation (min)		71	167	130	188	30.2	0.198	0.017	0.541

1 n = 8 replicate pens (6 birds/pen) for all treatments.

2 Coarse: hulls offered unground; Fine: hulls ground in a hammermill with a screen size of 1.0 mm.

3 GIT: gastrointestinal tract.

4 Phase segregation (min) presented as absolute values.

In the small intestine, the addition of AX led to a 17-min increase in MRT for digesta solids (*P* = 0.007) and a 22-min increase for digesta liquids (*P* < 0.001), while the particle size of SBH did not affect digesta MRT. In the hindgut, the addition of AX resulted in a prolonged MRT of liquid digesta (220 vs. 297 min, *P* = 0.020), causing a significant increase in digesta phase segregation of 68 min (*P* = 0.032). No interactions between particle size and AX were observed among treatments.

### Nutrient Digestibility and Retention

The addition of 50 g/Kg AX in the diet decreased the AID and ATTR of DM and starch (*P* < 0.05), regardless of the particle size of SBH (Table 5). The AID of N was reduced by 4%-points when AX was added to the diet with SBH-F but not with SBH-C (interaction, *P* = 0.024). Additionally, the ATTR of NSP increased by 2.4% units with SBH-F (*P* = 0.034) and by 4.3% units with the addition of AX (*P* < 0.001).

**Table 5:** Effect of arabinoxylan (AX) addition (50 g/Kg) to diets with different particle size of soybean hulls (SBH) on the apparent ileal digestibility (AID, %) and apparent total tract retention (ATTR, %) of dry matter (DM), nitrogen (N), starch, and total nonstarch polysaccharides (NSP) in broiler chickens.^1^^,^^2^

Particle size	Coarse		Fine		Pooled SEM	Particle size	AX	Particle size x AX
AX (g/Kg)	0	50	0	50				
AID								
DM	68.9	64.8	68.6	62.0	0.88	0.088	<0.001	0.165
Starch	96.2	95.1	96.2	94.6	0.18	0.185	<0.001	0.172
N	76.4^a^	75.1^a^	76.6^a^	72.3^b^	0.61	0.045	<0.001	0.024
ATTR								
DM	72.0	68.2	72.0	68.0	0.26	0.641	<0.001	0.608
Starch	97.6	96.9	97.7	97.0	0.09	0.258	<0.001	0.957
NSP	4.8	8.3	6.4	11.4	1.05	0.034	<0.001	0.484

a,b Means within a row lacking a common superscript differ (*P* < 0.05).

1 n = 8 replicate pens (6 birds/pen for AID, 9 birds/pen for ATTR) for all treatments.

2 Coarse: hulls offered unground; Fine: hulls ground in a hammermill with a screen size of 1.0 mm.

## DISCUSSION

The aim of this study was to elucidate how incorporating soluble, viscous fibers (AX) into diets containing iDF, in the form of SBH with different PS, affects digesta mean retention time and nutrient digestibility in broiler chickens. Our results reveal that the addition of AX modified some GIT traits, prolonged digesta MRT throughout the GIT, and reduced nutrient digestibility, mostly regardless of the PS of SBH. Moreover, PS of SBH only had an effect on NSP ATTR, increasing NSP degradation when SBH-F was used; and had no effects on GIT traits or digesta MRT. Finally, AID of N was reduced when adding AX to the diets with SBH-F but not with SBH-C.

In the current study, the addition of 50 g/Kg AX had a considerable impact on digestive processes in the proximal and distal GIT. In the proximal GIT, it reduced the relative weight of the gizzard and prolonged digesta MRT in the crop and the SI. The prolonged MRT coincided with reduced AID of nutrients as indicated by negative correlations between the MRT of liquid digesta in the SI and DM AID (r = −0.37, *P* = 0.036), N AID (r = −0.36, *P* = 0.036), and starch AID (r = −0.34, *P* = 0.060). This correlation illustrates that prolonged MRT is not inherently associated to improved nutrient digestibility. Previous studies have similarly reported that the addition of sDF into the diets of chickens (van der Klis and van Voorst, 1993) and pigs (Schop et al., 2020) prolongs digesta MRT and reduces nutrient digestibility (Choct and Annison, 1992; Langhout et al., 2000; Lin and Wright, 2018). These phenomena can be explained by the increase in digesta viscosity that arises in the presence of sDF (Bedford and Classen, 1993; Carré et al., 1994; Chen et al., 2020). The rise in digesta viscosity has been associated with reduced contractile activity (Smulikowska et al., 2002), leading to a slower digesta transit. Moreover, elevated viscosity of the digesta imposes greater restrictions on the enzyme-substrate interaction (Lentle and de Loubens, 2015), resulting in changes in mixing, diffusion, and absorption of nutrients; ultimately, affecting overall digestion efficiency (Smits et al., 1997). In such circumstances, it is plausible to attribute the lower values of DM AID and starch AID to the structural and chemical impairments caused by the AX added.

Interestingly, our findings indicate that the addition of AX into the diets led to a reduction in N AID by 4.3%-units only in the presence of SBH-F. The fact that we did not see effects of PS on AID of N for the diets without AX, suggests that AX affects the digestibility of proteins from ingredients other than SBH. Moreover, if the digestibility of SBH proteins was different between SBH-F and SBH-C diets, we would expect the SBH-F to have higher AID of N compared with SBH-F, not lower. One potential explanation for this phenomenon may be linked to the interplay between the particles and the viscosity of the digesta. In model studies, it has been reported that the presence of particles can modulate the viscosity of a suspension, depending on the viscosity of the liquid phase of the suspension and the particle size (Senapati et al., 2010; Konijn et al., 2014). Therefore, it is reasonable to speculate that SBH-C may mitigate the adverse effects of AX on digesta viscosity compared to SBH-F. This reduction in N AID then might be related to a digesta matrix effect, to the hindrance of enzymes, or to incomplete absorption.

In the distal GIT, the addition of AX also significantly affected the length and weight of the ceca, digesta transit behavior, and nutrient ATTR-AID (disappearance in hindgut). The ceca play a crucial role in chickens for nutrient fermentation and fermentation-products absorption (Svihus et al., 2013). As a positive correlation was observed between nutrient disappearance in the hindgut, NSP ATTR, and ceca traits (Table 6), it is possible to suggest that enhanced fermentation processes may promote development of the ceca.

**Table 6:** Pearson correlation coefficients of nutrient disappearance in the hindgut (ATTR-AID), NSP ATTR, and ceca traits.^1^^,^^2^^,^^3^

Parameters	DM disappearance	Starch disappearance	NSP ATTR	Ceca RW	Ceca length
DM disappearance	1.00				
Starch disappearance	0.75*	1.00			
NSP ATTR	0.47*	0.52*	1.00		
Ceca RW	0.34†	0.43*	0.57*	1.00	
Ceca length	0.28	0.48*	0.45*	0.81*	1.00

1 Parameters n = 32 replicate pens (6 or 9 birds/pen).

2 ATTR: apparent total tract retention; AID: apparent ileal digestibility; NSP: non-starch polysaccharides.

3 * *P* ≤ 0.05; †*P* ≤ 0.10.

**Figure 1:**
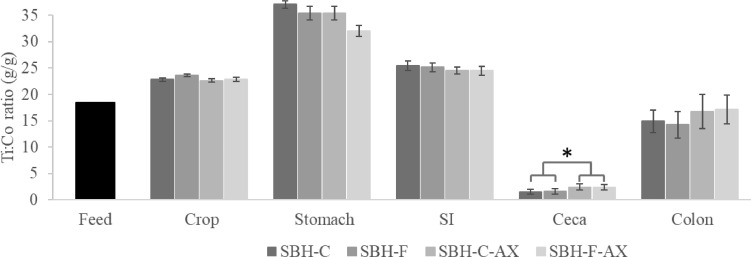
Titanium:cobalt ratio in feed, crop, stomach (proventriculus plus gizzard), SI (small intestine), ceca, and colon from broiler chickens fed diets containing soybean hulls (SBH) with or without arabinoxylans (AX). SBH-C: coarse SBH without AX; SBH-F: fine SBH without AX; SBH-C-X: coarse SBH with AX; SBH-F-AX: fine SBH with AX. Error bars indicate SEM. Asterisks indicates differences among diets within segment (*P < 0.05).

Furthermore, our results suggest that addition of AX may impact the flow of solids and/or liquids into or out of the ceca. We observed that limited amount of solids (represented by Ti) entered the ceca (2.96 mg in the ceca vs. 6.17mg in the colon), regardless of viscosity and fiber particle size. Other studies (Vergara et al., 1989; Rougière and Carré, 2010; de Vries et al., 2014; Garçon et al., 2023) have reported similar observations. Nevertheless, insoluble marker:soluble marker ratios were significantly higher in AX diets (1.09 vs. 1.66 g/g, *P* < 0.001), indicating that there is a reduced digesta phase separation in the ceca with these diets (Figure 1). It is possible that the high viscosity of the digesta influences cecal mixing and emptying processes (Clench, 1999). Further investigation is necessary to better understand the underlying factors influencing cecal entry and evacuation behavior of solid and liquid digesta.

The decrease in PS of SBH resulted in greater NSP ATTR, but it had no effect on GIT traits, digesta transit, and nutrient AID. The improvement in NSP ATTR could be explained by the increase in surface area that occurs when larger fiber particles are ground, facilitating more effective microbial colonization (Wong and Jenkins, 2007). This improved accessibility subsequently promotes NSP fermentation by gut microbiota. Although SBH are scarcely studied (Griffith, 1969; Tejeda and Kim, 2021), several studies have demonstrated an effect of PS of iDF on proventriculus (Jiménez-Moreno et al., 2011), gizzard (Hetland and Svihus, 2001; Hetland et al., 2003, 2005; Sacranie et al., 2012) and small intestine (Jørgensen et al., 1996) development. Also, it has been reported that PS of iDF affects digesta passage rate (Svihus et al., 2002). However, most of these findings are in relation to iDF other than SBH. Some of these materials, like oat hulls, can be considered rather hard or resistant to grinding. The harder the iDF, the longer the retention time of the material in the gizzard, as observed by others (Ferrando et al., 1987; Nishii et al., 2016). Another possible reason for the lack of PS effects on digesta transit could be related to the insoluble marker used to represent the particulate digesta. Given that titanium dioxide has a small particle size, it may not accurately reflect the retention of the whole digesta, but rather mainly the retention of fine particles, as noted by Svihus et al. (2002). This underlines the importance of using mordanted markers, particularly when using fibrous diets, as done by others (Ferrando et al., 1987; Solà-Oriol et al., 2010; Dorado-Montenegro et al., 2023), to track the transit behavior of the iDF.

In conclusion, adding sDF, in the form of purified wheat AX, considerably influenced digestive processes in the proximal and distal GIT. In the proximal GIT, addition of AX led to a decrease in the RW of the gizzard, prolonged MRT of digesta in the crop and small intestine, and reduced nutrient digestibility. In the hindgut, addition of AX increased ceca size and prolonged MRT of digesta; coinciding with increased disappearance of starch. In addition, our data suggest that addition of AX may influence filling or emptying processes of the ceca, and increased retention of solids and liquids in the hindgut. Reducing particle size of SBH increased ATTR of NSP by 4.3%-units but had no impact on GIT development, digesta MRT, or digestion of other nutrients. Furthermore, the addition of AX reduced AID of N only in the presence of SBH-F, but not SBH-C. This suggests that coarse particles may, to some extent, counteract the negative effects of soluble, viscous, fibers on nutrient digestion.

### Declaration of AI and AI-Assisted Technologies in the Writing Process

During the preparation of this work the author(s) used ChatGPT/Open AI in order to improve readability of the text and language. After using this tool/service, the author(s) reviewed and edited the content as needed and take(s) full responsibility for the content of the publication.
